# Bedside Ultrasound Versus Computed Tomography in Adult Neutropenic Patients with Acute Abdominal Symptoms: A Comparative Study

**DOI:** 10.3390/diagnostics16132059

**Published:** 2026-07-01

**Authors:** Maria Costanza Caparello, Salvatore Massimo Stella, Riccardo Morganti, Emilia Bramanti, Chiara Arena, Francesca Cerri, Katia Valentini, Luigi De Simone, Sara Galimberti, Edoardo Benedetti

**Affiliations:** 1 Department of Clinical and Experimental Medicine, UO Haematology, Azienda Ospedaliero-Universitaria Pisana, 56127 Pisa, Italy; maria.costanza87@gmail.com (M.C.C.); sara.galimberti@med.unipi.it (S.G.); 2 Società Italiana di Ultrasonografia in Medicina e Biologia (SIUMB), Via dei Gracchi 278, 00192 Roma, Italy; smstella@alice.it; 3 Section of Statistics, Azienda Ospedaliero-Universitaria Pisana, 56127 Pisa, Italy; r.morganti@ao-pisa.toscana.it; 4 Institute of Chemistry of Organo Metallic Compounds (ICCOM), CNR, Via G Moruzzi 1, 56124 Pisa, Italy; emilia.bramanti@cnr.it; 5 Dipartimento di Radiologia, Azienda Ospedaliero-Universitaria Pisana, 56127 Pisa, Italy; chiaraarena@yahoo.it (C.A.); francerri@gmail.com (F.C.); 6 Anesthesia and Maternal-Infantile Resuscitation Unit, Azienda Ospedaliero Universitaria Pisana, 56127 Pisa, Italy; k.valentini@hotmail.it (K.V.); l.desimone@ao-pisa.toscana.it (L.D.S.)

**Keywords:** neutropenic enterocolitis (NEC), bedside ultrasound (BS-US), computed tomography (CT), bowel wall thickness, acute abdominal pain, hematological patients

## Abstract

**Background:** Abdominal pain in hematological patients, particularly during chemotherapy-induced neutropenia, represents a significant diagnostic challenge due to the broad spectrum of potentially life-threatening conditions, including neutropenic enterocolitis (NEC). Computed tomography (CT) is considered the reference imaging modality; however, its use is limited by radiation exposure, and the need for patient transport. Bedside ultrasound (BS-US) may offer a rapid, non-invasive, and repeatable alternative. **Methods:** This prospective study compared BS-US and CT in 65 hematological patients presenting with acute abdominal pain. Concordance between the two modalities was evaluated in terms of intestinal site localization, bowel wall thickness (BWT), and final diagnosis. Diagnostic agreement was assessed using Cohen’s kappa coefficient, and additional diagnostic accuracy metrics—including sensitivity, specificity, positive predictive value, and negative predictive value—were calculated. BWT measurements were analyzed using Bland–Altman methods. **Results:** A high level of agreement was observed between BS-US and CT in both intestinal localization and final diagnosis. Agreement for intestinal site localization was good (Cohen’s κ = 0.964), as was diagnostic concordance (Cohen’s κ = 0.962), and using CT as the reference standard, BS-US showed uniformly good diagnostic performance across all evaluated conditions, with sensitivity, specificity, PPV, and NPV consistently reaching 1.00 and confirming strong agreement between BS-US and CT. These findings were consistent across different clinical settings (hematology unit and Intensive Care Unit) and independent of body mass index. In NEC cases, BWT measurements showed strong concordance between CT and BS-US, with only 4.6% of values outside the limits of agreement in Bland–Altman analysis. **Conclusions:** BS-US demonstrated a good agreement with CT and proved to be a reliable, safe diagnostic tool in hematological patients with acute abdominal pain. These findings indicate that bedside ultrasound represents a valuable and safe diagnostic tool in neutropenic hematological patients with acute abdominal pain, providing crucial information in a clinically fragile population that may not always be suitable for CT due to their unstable condition. While our study is hypothesis-generating, the role of BS-US in this setting emerges as a reasonable, evidence-supported hypothesis that warrants further prospective evaluation.

## 1. Introduction

Abdominal pain in hematological patients undergoing intensive chemotherapy—particularly during prolonged neutropenia—represents a major diagnostic challenge, as it may signal severe and potentially life-threatening complications. Among these, neutropenic enterocolitis (NEC) [1,2,3,4,5], or typhlitis, is one of the most feared gastrointestinal emergencies. NEC is characterized by fever, abdominal pain, diarrhea, and bowel wall thickening occurring in the setting of chemotherapy-induced neutropenia, most frequently in patients with hematological malignancies receiving intensive treatment or stem cell transplantation [6,7,8,9]. Reported incidence varies widely (0.8–26%), and mortality historically reached 30–50%, particularly when diagnosis is delayed [1,2].

Early recognition is therefore crucial. In a large prospective study of 1754 hematological patients, Benedetti et al. demonstrated that bedside ultrasound (BS-US) enabled prompt NEC diagnosis and reduced mortality to 9.4%. In that study, bowel wall thickening (BWT) ≥4 mm detected by ultrasound, combined with fever, abdominal pain, or diarrhea, represented the major diagnostic criterion for NEC [1,4,8,9,10].

CT has traditionally been considered the reference imaging modality for evaluating abdominal pain in neutropenic patients due to its panoramic visualization, high reproducibility, and ability to detect complications such as perforation, ischemia, abscesses, or extensive intestinal involvement. However, CT also presents important limitations in this fragile population: the need for patient transport, exposure to ionizing radiation, frequent use of intravenous contrast with potential nephrotoxicity, and substantial logistical and economic burden [6,7,9,11]. These issues are particularly relevant in hematological patients who often require repeated imaging and may present with clinical instability, thrombocytopenia, renal impairment, or profound immunosuppression.

In contrast, BS-US offers a non-invasive, radiation-free, repeatable, and immediately available alternative that can be performed directly at the patient’s bedside. Several studies have shown that ultrasound can accurately detect bowel wall thickening, intestinal distension, free fluid, and inflammatory changes, making it especially useful in the early evaluation of acute abdominal pain [1,9]. In abdominal emergencies, ultrasound provides approximately 40% more diagnostic information than clinical examination alone and changes management in nearly 20% of cases, while reducing the need for emergency CT scans [12,13].

Bowel wall thickness is the cornerstone imaging parameter in both inflammatory [14] and neutropenic bowel disease [1,2,3,5]. Early detection of BWT may represent the earliest objective sign of NEC before clinical deterioration becomes evident.

Despite growing recognition of the value of BS-US, CT remains widely regarded as the standard imaging technique in hematological patients with acute abdominal pain [15,16]. However, direct comparative data between BS-US and CT in this specific clinical setting remain limited [12,13].

The aim of the present study was therefore to compare BS-US with CT in hematological patients presenting with acute abdominal pain, assessing the degree of agreement between the two modalities. Specifically, the study sought to: (i) compare CT and BS-US in identifying the abdominal site responsible for symptoms; (ii) in cases of intestinal involvement, evaluate agreement regarding the intestinal segment affected, the presence and extent of BWT (including BWT measurements in millimeters), and the final diagnosis (including NEC, intestinal distension [17,18], chemotherapy-related diarrhea, diverticulitis, appendicitis, lymphoma involvement, and other causes of abdominal pain).

Our hypothesis was that BS-US could provide clinically relevant diagnostic information just as CT, in neutropenic hematological patients, reserving CT for inconclusive cases, suspected complications, or a broader differential diagnosis.

## 2. Materials and Methods

### 2.1. Study Design and Patients

From May 2020 to December 2025, we prospectively enrolled 65 consecutive adult patients affected by hematological malignancies presenting with acute abdominal symptoms during chemotherapy-induced neutropenia at the University of Pisa.

Eligible patients were all neutropenic hematological patients who underwent abdominal computed tomography (CT) for clinical decision-making due to the onset of abdominal symptoms, including abdominal pain with or without diarrhea, with or without fever, and with or without abdominal distension.

All patients were neutropenic due to chemotherapy and were classified as grade 3 neutropenia (absolute neutrophil count [ANC] < 1.0 × 10^9^/L) or grade 4 neutropenia (ANC < 0.5 × 10^9^/L).

Patients were enrolled either during admission to the Hematology and Bone Marrow Transplant Unit (Hema/BMT) or, in cases of clinical deterioration, after transfer to the Intensive Care Unit (ICU). Both units are located within Santa Chiara University Hospital, Pisa, Italy. Accordingly, abdominal CT scans were requested either by hematologists in the Hema/BMT Unit or by ICU physicians after transfer to intensive care.

Abdominal CT was performed within 12 h from the onset of clinical symptoms. Both CT and BS-US were available 24 h a day, 7 days a week, including weekends.

In accordance with established recommendations for neutropenic patients with abdominal pain, no confirmatory biopsies were performed in our cohort due to the patients’ frail clinical condition and the well-recognized risks associated with invasive procedures in this setting. As highlighted by Gorschlüter et al. [4,8], histologic confirmation is often not feasible or clinically informative in neutropenic or thrombocytopenic patients, in whom endoscopic procedures carry a substantial risk of bleeding or perforation. Consistent with this approach, biopsies were obtained only in allogeneic transplant recipients with a specific clinical suspicion of gastrointestinal GVHD, in whom rectal biopsy confirmed the diagnosis. These 3 patients were affected only by intestinal GVHD. No biopsies were performed in patients evaluated for suspected NEC, for whom diagnosis relied on established clinical and radiological criteria using CT or bedside ultrasound, as also reported by Benedetti et al. [1].

### 2.2. Bedside Ultrasound Examination

Within 24 h after abdominal CT (Abd-CT), all patients underwent BS-US, performed by a hematologist who was a faculty member and teacher at the Italian School of Basic and Emergency Ultrasound (SIUMB—Italian Society of Medical and Biological Ultrasonography), University of Pisa, with specific expertise in gastrointestinal ultrasound (GIUS) [1,6,7]. The operator was blinded to CT findings.

Abdominal CT was considered the reference standard imaging modality, while BS-US represented the investigational arm of the study.

Both abdominal ultrasound and intestinal ultrasound were performed according to previously published recommendations [1,4,11]. Images were systematically obtained from the caecum, ascending colon, transverse colon, descending colon, ileum, jejunum, and appendix when assessable.

All ultrasound images were digitally stored and independently reviewed by two SIUMB-certified physicians blinded to CT results and blinded to each other’s reports.

### 2.3. Ultrasound Equipment

Bedside ultrasound was performed using either Esaote Class C Advance or General Electric (GE) Logiq E10s (GE HealthCare, Milwaukee, WI, USA). For abdominal and intestinal scanning, convex probes were used: 2–5 MHz bandwidth (GE) or 3.5–5 MHz convex probe (Esaote, Florence, Italy). For high-resolution intestinal assessment, linear probes were used: 4–16 MHz linear matrix array probe (GE) or 7.5 MHz linear probe (Esaote). The intestinal tract was scanned according to previously described GIUS protocols [1,11,19].

### 2.4. CT and Image Review

One experienced sonographer performed all ultrasound examinations, and two additional certified sonographers independently reviewed the stored ultrasound images in a blinded fashion. Thus, a total of three sonographers contributed to the ultrasound component: one for image acquisition and two for image interpretation.

An identical structure was applied to CT: one radiologist performed and reported the CT examination, and two additional radiologists independently reviewed the CT images in a blinded manner. Therefore, three radiologists contributed to the CT evaluation: one for acquisition/reporting and two for blinded review.

CT scan was performed using thin slice acquisitions of 2.5 mm (reconstructed at 1.25 mm) before and after intravenous administration of iodinated contrast medium, during the arterial and venous phases. All imaging findings were recorded in dedicated study files inaccessible to physicians not participating in the study. Patient-sensitive data were encrypted, and a numerical identification code was assigned to each participant to ensure confidentiality.

### 2.5. Study Endpoints and Comparative Analysis

For each patient, we analyzed concordance (CC) and discordance (DC) between abdominal CT and BS-US regarding:

1. Abdominal Site Involved
  Identification of the abdominal organ responsible for the symptoms reported by the patient.
2. Intestinal Involvement
  When the intestine was involved, we compared:
  - Intestinal Site Involved
    We localized the pathological intestinal segment, including colon, ileum, jejunum, appendix, and combined intestinal segments.
  - Intestinal Distension
    When intestinal distension was present, the maximal diameter of the colon and/or ileum was measured in centimeters from serosa to serosa, according to EFSUMB recommendations [19].
  - Bowel Wall Thickness (BWT)
    When bowel wall thickening was present, bowel wall thickness was measured in millimeters from the serosal surface to the mucosal–lumen interface, according to previously validated criteria.
    In cases of suspected neutropenic enterocolitis (NEC), diagnosis was established according to previously published criteria, defining NEC as bowel wall thickening ≥4 mm associated with neutropenia and compatible clinical symptoms [1,4,6,7,8].
3. Intestinal Content
  The intestinal lumen content was classified as: liquid, solid, or mixed liquid-solid [1,4,8,19].
4. Intestinal Motility
  Bedside ultrasound also assessed bowel motility, which was systematically recorded [1,2,4,20].
5. Final Diagnosis
  Diagnostic agreement between CT and BS-US was evaluated for the final diagnosis, including neutropenic enterocolitis (NEC), chemotherapy-related diarrhea (CHT-D), intestinal distension, paralytic ileus, intestinal involvement by lymphoma, appendicitis, diverticulitis, and perforation.

### 2.6. Negative Imaging Findings

Patients with normal CT findings, normal BS-US findings, or both were also included, and the absence of pathological findings was recorded.

### 2.7. Statistical Analysis

Categorical data were described using absolute and relative frequencies (%), while continuous variables were summarized as mean ± standard deviation.

Agreement between CT and BS-US for categorical variables such as diagnosis, intestinal site involved, and distension site was assessed using Cohen’s kappa coefficient.

Agreement for continuous variables such as bowel wall thickness was evaluated using Bland–Altman analysis. All statistical analyses were performed using SPSS version 29.

Diagnostic performance metrics, including sensitivity, specificity, positive_and negative predictive_value (PPV and NPV).

### 2.8. Ethical Approval

All patients provided written informed consent before enrollment.

The study was approved by the local Ethics Committee (Institutional Review Board n° 12184 approved on 27 February 2020).

## 3. Results

Patients’ characteristics are summarized in Table 1.

A good agreement was observed between CT and BS-US in identifying both the abdominal site involved and the final diagnosis. In cases of NEC, a strong concordance was found between CT and BS-US in assessing bowel wall thickness.

**Figure 1:**
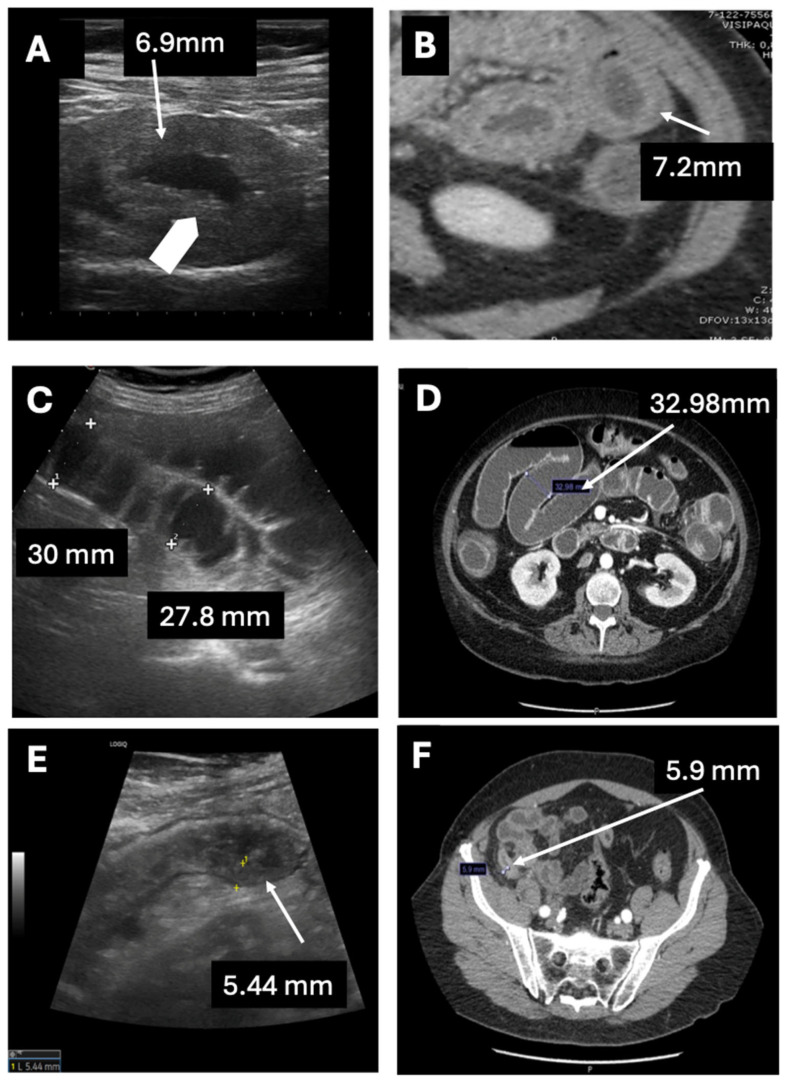
Comparison between CT and BS-US in the diagnosis of NEC (panel **A** and **B**), abdominal distention (panel **C** and **D**) and appendicitis (panel **E** and **F**). (**A**) BS-US showing NEC with BWT 6.9 mm; (**B**) CT showing BWT 7.22 mm; white arrowheads show a microbubble of air within injured bowel wall visible with both BS-US and CT. (**C**) BS-US showing intestinal distension with fluid filled content (30 mm and 27.8 mm); (**D**) same images in CT (32.98 mm); (**E**) BS-US showing acute appendicitis (5.44 mm); (**F**) same image with CT (BWT 5.9 mm).

**Figure 2:**
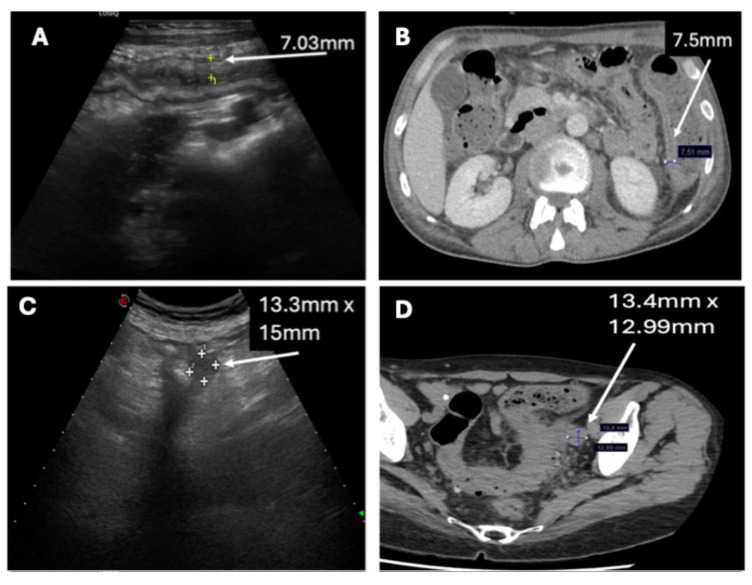
Comparison between CT and BS-US in the diagnosis of NEC (panels **A** and **B**) and diverticulitis (panels **C** and **D**). (**A**) BS-US showing NEC with BWT (7.03 mm); (**B**) CT showing NEC with BWT 7.5 mm. (**C**) BS-US showing diverticulitis with BWT (13.3 mm × 15 mm); (**D**) CT showing diverticulitis with BWT (13.4 mm × 12.99 mm).

Figure 1 illustrates the agreement between CT and BS-US in the diagnosis of NEC (panel A and B), as well as in other conditions such as abdominal distention (panel C and D), and appendicitis (panel E and F). Similarly, Figure 2 shows concordance between CT and BS-US in the diagnosis of NEC (panel A and B) and diverticulitis (panel C and D).

**Figure 3:**
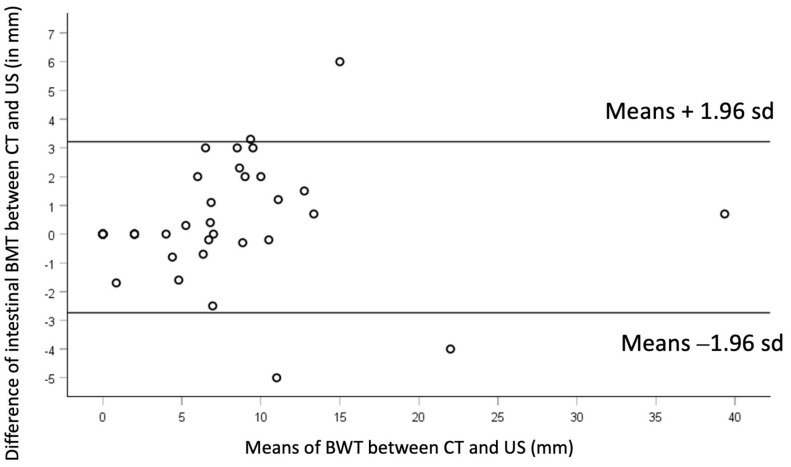
Bland–Altman plot showing the difference in mean of BWT between CT and BS-US (mm).

Focusing on NEC cases, the measurements of bowel wall thickness (expressed in millimeters) obtained by CT and BS-US were highly consistent. This agreement is quantitatively reported in Figure 3, where a Bland–Altman analysis was performed to assess the agreement between CT and BS-US in measuring bowel wall thickness.

The Bland–Altman analysis was based on 65 paired CT–US measurements of BWT. The mean bias between CT and BS US was 0.238 mm, with limits of agreement ranging from −2.8 mm to +3.2 mm (mean ± 1.96 SD). Only three of the 65 measurements (4.6%) fell outside these limits, confirming a high level of agreement between the two modalities.

Table 2 reports the agreement and disagreement between BS-US and CT in identifying the intestinal site involved in the patient population. A very high level of concordance was observed between the two imaging modalities, as demonstrated by a Cohen’s kappa coefficient of 0.964, indicating excellent agreement.

Table 3 presents the concordance and discordance between BS-US and CT with respect to the diagnosis that led to the indication for the CT scan. Also in this case, the agreement between the two techniques was very strong, with a Cohen’s kappa coefficient of 0.962, reflecting very good agreement in diagnostic accuracy.

Table 4 summarizes the agreement between BS-US and CT considering both the intestinal site involved and the final diagnosis, with additional stratification based on clinical setting (hematology unit vs. Intensive Care Unit) and body mass index (BMI). The results show that the high level of concordance between the two imaging methods was maintained irrespective of patient location an BS-US machine used (hematology unit vs. ICU) and BMI categories (<25 vs. ≥25). This confirms the robustness and reliability of BS-US compared to CT across different clinical conditions and patient characteristics.

Using CT as the reference standard, ultrasound achieved a good diagnostic performance for all intestinal sites. Sensitivity, specificity, PPV, and NPV were 1.00 for colon (95% CI: 0.88–1.00, 0.90–1.00, 0.88–1.00, 0.90–1.00), ileum and jejunum (95% CI: 0.81–1.00, 0.92–1.00, 0.81–1.00, 0.92–1.00), and ileum and colon (95% CI: 0.79–1.00, 0.93–1.00, 0.79–1.00, 0.93–1.00).

Using CT as the reference standard, ultrasound demonstrated good diagnostic performance across all conditions. Sensitivity, specificity, PPV, and NPV were all 1.00 for appendicitis (95% CI: 0.21–1.00; 0.94–1.00; 0.21–1.00; 0.94–1.00), chemotherapy-related diarrhea (95% CI: 0.72–1.00; 0.93–1.00; 0.72–1.00; 0.93–1.00), colon–ileum distension (95% CI: 0.16–1.00; 0.94–1.00; 0.16–1.00; 0.94–1.00), diverticulitis (95% CI: 0.29–1.00; 0.94–1.00; 0.29–1.00; 0.94–1.00), heteroplasia (95% CI: 0.21–1.00; 0.94–1.00; 0.21–1.00; 0.94–1.00), intestinal GVHD (95% CI: 0.29–1.00; 0.94–1.00; 0.29–1.00; 0.94–1.00), paralytic ileus (95% CI: 0.59–1.00; 0.94–1.00; 0.59–1.00; 0.94–1.00), intestinal lymphoma involvement (95% CI: 0.72–1.00; 0.93–1.00; 0.72–1.00; 0.93–1.00), and neutropenic enterocolitis (95% CI: 0.87–1.00; 0.91–1.00; 0.87–1.00; 0.91–1.00). These results consistently confirm the agreement between BS-US and CT across all diagnostic categories.

## 4. Discussion

The present study demonstrates a good level of agreement between bedside ultrasound (BS-US) and computed tomography (CT) in neutropenic hematological patients presenting with acute abdominal pain. BS-US accurately identified the intestinal site involved, reproduced bowel wall thickness (BWT) measurements with high precision, and achieved very strong diagnostic concordance with CT across all major clinical conditions, including neutropenic enterocolitis (NEC) [1,2,3,4,5], intestinal distension, chemotherapy-related diarrhea, diverticulitis, and appendicitis [13,21,22]. Among these, NEC remains one of the most feared complications due to its high morbidity and historically elevated mortality rates, reaching up to 30–50% in delayed diagnoses [1]. Early recognition and prompt treatment are therefore crucial to improve outcomes [9].

BS-US is a rapid, repeatable, non-invasive, radiation-free, and cost-effective imaging modality that can be performed immediately at the patient’s bedside, making it particularly attractive in hematology units and intensive care settings [1,7,9,23].

Compared with the previous literature, our findings (Figure 1 and Figure 2) reinforce and expand the evidence supporting the diagnostic value of ultrasound in this fragile population. Earlier studies [1,4,7,8,9,10,24] have shown that BS-US can detect early bowel wall abnormalities in neutropenic patients and may facilitate timely diagnosis of NEC, but direct, systematic comparisons with CT have been limited. Our prospective dataset of 65 consecutive patients (Table 2 and Table 3) provides one of the most robust head-to-head evaluations to date, confirming that BS-US can reproduce CT findings with remarkable accuracy, including in challenging settings such as the ICU and in patients with higher BMI. The high concordance in BWT measurements, with only 4.6% of values outside Bland–Altman limits, further supports the reliability of BS-US for quantitative assessment. These findings are particularly important because precise anatomical localization directly influences diagnostic suspicion and therapeutic decisions [12].

The most frequent causes of abdominal pain in neutropenic patients were neutropenic enterocolitis (NEC; 27/65 total, 26 by CT and 27 by BS-US), followed by intestinal involvement by lymphoma (*n* = 11), chemotherapy-related disease (CHT-D; *n* = 10) [25], and paralytic ileus (*n* = 7) [24,26,27].

These data demonstrate that BS-US is able not only to identify bowel abnormalities but also to provide the same diagnostic categorization as CT, supporting clinical decision-making with high reliability [12,13].

The observation that CT–BS-US concordance remains high even in the ICU subgroup (Table 4) is clinically meaningful, as it suggests that BS-US may remain feasible and reliable also in critically ill neutropenic patients, including those transferred to the ICU due to clinical deterioration. Thus, our findings suggest that BS-US may represent a useful imaging option in fragile neutropenic patients, particularly those whose clinical condition or degree of immunosuppression makes transport to the radiology department—and especially repeated CT examinations—more challenging [1,7,9,13,21,22].

We acknowledge that an “ultrasound-first strategy” [12,13], although increasingly adopted in clinical practice, presents several intrinsic limitations that must be carefully considered when interpreting our findings. First, intestinal ultrasound is operator-dependent, and its diagnostic accuracy is ensured only when examinations are performed by a well-trained physician with specific expertise in bowel imaging; this requirement may limit the generalizability of our results to centers where such expertise is not consistently available. Furthermore, despite its strengths in assessing luminal disease activity, ultrasound offers only a limited evaluation of extraintestinal complications, which may necessitate complementary imaging modalities to achieve a comprehensive assessment. Collectively, these limitations highlight the need for further standardization, multicenter prospective studies, and integrated diagnostic pathways to fully define the role of ultrasound-first strategies in routine care.

The concordance observed across different clinical settings is particularly relevant. Recent prospective data show that NEC represents a clinically significant complication not only after alloSCT but also in patients undergoing autoSCT or intensive chemotherapy [6,7]. Both alloSCT and autoSCT+CHT recipients are typically managed in single rooms with low microbial burden and represent highly fragile populations in whom rapid bedside assessment is essential. In this context, the high agreement between CT and BS-US observed in our cohort across these settings suggests that BS-US may provide timely and clinically meaningful information regardless of transplant type or treatment pathway, including in patients who are difficult to transport for CT due to their clinical instability.

This study also has limitations. The sample size, although larger than in most previous comparative studies, remains modest and may limit subgroup analyses. BS-US examinations were performed by an operator with specific expertise in gastrointestinal ultrasound, which may affect generalizability to centers with less experience. Additionally, although CT was used as the reference standard, histological confirmation was not feasible in this population due to clinical contraindications, consistent with current practice in neutropenic patients [8].

Despite these limitations, our study adds novel evidence to the literature by providing a prospective, systematically collected comparison between BS-US and CT in a real-world cohort of neutropenic hematological patients. The high concordance observed across multiple diagnostic domains supports the integration of BS-US into routine evaluation of acute abdominal symptoms in this setting and highlights its potential role in improving diagnostic timeliness and patient safety.

## 5. Conclusions

Our study demonstrates that BS-US provides comparable morphological information relevant to NEC assessment to CT in adult hematological patients with acute abdominal pain regarding intestinal site involved, final diagnosis, intestinal distension, and bowel wall thickness assessment in NEC. Given its safety profile, bedside availability, repeatability, and strong diagnostic performance, ultrasound should be considered the preferred first-line imaging modality, reserving CT for inconclusive cases, complications, or broader differential diagnosis. This ultrasound-first strategy may improve diagnostic timing, reduce unnecessary radiation exposure, lower healthcare costs, allow close follow-up, and ultimately improve outcomes in this highly vulnerable patient population.

## Figures and Tables

**Table 1:** Characteristics of the population. Statistics: mean (sd) or frequency (%).

Characteristics	Statistics
**Gender**	
M	36 (55.4)
F	29 (44.6)
**Age: median (range)**	49 (22–85)
**BMI categories**	
<25	38 (58.5)
≥25	27 (41.5)
**Hematology**	
No (ICU)	23 (35.4)
yes	42 (64.6)
**Diagnosis**	
ALL	6 (7.7)
CLL	1 (1.5)
AML	36 (46.1)
NHL	22 (33.9)
MF	1 (1.5)
HD	2 (3.1)
MM	4 (6.2)
**Therapy**	
Allogenic Tx	12 (18.5)
ASCT	3 (4.6)
CHT	50 (61.6)

Legend: M = male; F = female; BMI = body mass index; ALL = Acute Lymphoblastic Leukemia; CLL = chronic lymphocytic leukemia; AML = Acute Myeloid Leukemia; NHL = non-Hodgkin lymphoma; MF = primary myelofibrosis; HD = Hodgkin lymphoma; MM = multiple myeloma; Allogenic Tx = allogeneic stem cell transplant; ASCT = autologous stem cell transplant; CHT = chemotherapy.

**Table 2:** Agreement analysis between BS-US and CT related to intestinal site. Cohen’s kappa equal to 0.964.

	**CT Scan**					
**BS-US Scan**		**Colon**	**Ileum and Jejunum**	**Appendix**	**Ileum and Colon**	**Total**
	**Colon**	30	0	0	0	30
	**Ileum and jejunum**	0	18	0	0	18
	**Appendix**	0	0	1	0	1
	**Ileum and colon**	0	0	0	16	16
	**Total**	30	18	1	16	65

**Table 3:** Agreement analysis between BS-US and TC related to diagnosis. Cohen’s kappa equal to 0.962 (very good agreement).

**CT Scan**											
		**Appendicitis**	**CHT-D**	**Colon–Ileum Dis**	**Diverticulitis**	**Heteroplasia**	**GVHD**	**PI**	**Disease**	**NEC**	**Total**
**BS-US Scan**	**Appendicitis**	1	0	0	0	0	0	0	0	0	1
	**CHT-D**	0	10	0	0	0	0	0	0	0	10
	**Colon–Ileum Dis**	0	0	2	0	0	0	0	0	0	2
	**Diverticulitis**	0	0	0	3	0	0	0	0	0	3
	**Heteroplasia**	0	0	0	0	1	0	0	0	0	1
	**GVHD**	0	0	0	0	0	3	0	0	0	3
	**PI**	0	0	0	0	0	0	7	0	0	7
	**Disease**	0	0	0	0	0	0	0	11	0	11
	**NEC**	0	1	0	0	0	0	0	0	26	27
	**Total**	1	11	2	3	1	3	7	11	26	65

Legend: CHT-D = chemotherapy related diarrhea. Colon–Ileum Dis: Colon and/or Ileum distended. Heteroplasia = adenocarcinoma of the descending colon. GVHD: graft versus host disease (intestinal). PI = paralytic ileus. Disease = intestinal involvement by lymphoma. NEC = neutropenic enterocolitis.

**Table 4:** Cohen’s kappa values stratified for hematology/ICU and BMI <25/≥25.

Factor	ICU	Hematology	BMI < 25	BMI ≥ 25
Diagnosis	0.943	0.971	1.0	0.907
Intestinal site	0.898	1.0	1.0	0.916

## Data Availability

The original contributions presented in this study are included in the article. Further inquiries can be directed to the corresponding author.

## Abbreviations

The following abbreviations are used in this manuscript:

BMI	Body Mass Index
CHT-D	Chemotherapy-related diarrhea
Colon–Ileum Dis	Colon and/or ileum distended
PI	Paralytic ileus
GVHD	Graft versus host disease (intestinal)
NEC	Neutropenic enterocolitis
TrC	Trasverse colon
AC	Ascending colon
DC	Descending colon
J	Jejunum
I	Ileum
App	Appendicitis
BWT	Bowel wall thickening
CT	Computed tomography
ICU	Intensive Care Unit
BS-US	Bedside ultrasound
M/F	Male/female
MF	Primary myelofibrosis
AML	Acute myeloid leukemia
ALL	Acute lymphoblastic leukemia
CLL	Chronic lymphocytic leukemia
CHT	Chemotherapy
ASCT	Autologous stem cell transplant
Allogenic Tx	Allogeneic stem cell transplant
MM	Multiple myeloma
HD	Hodgkin lymphoma
NHL	Non-Hodgkin lymphoma
